# Agreeing Language in Veterinary Endocrinology (ALIVE): Hypothyroidism, Hyperthyroidism, (Euglycaemic) Diabetic Ketosis/Ketoacidosis, and Diabetic Remission—A Modified Delphi-Method-Based System to Create Consensus Definitions

**DOI:** 10.3390/vetsci13010035

**Published:** 2026-01-01

**Authors:** Stijn J. M. Niessen, Robert Shiel, Astrid Wehner, Miguel Campos, Sylvie Daminet, Federico Fracassi, Peter Graham, Jérémie Korchia, Patty Lathan, Rodolfo Oliveira Leal, Diego Daniel Miceli, Carmel T. Mooney, Maria de los Doloros Perez Alenza, Mark E. Peterson, Johan P. Schoeman, on behalf of the ESVE/SCE membership

**Affiliations:** 1 Royal Veterinary College, University of London, London AL9 7TA, UK; 2 Veterinary Information Network, Davis, CA 95616, USA; 3 Veterinary Specialist Consultations, 1215 JX Hilversum, The Netherlands; 4 Department of Veterinary Clinical Sciences, Faculty of Veterinary Medicine, Michigan State University, East Lansing, MI 48824, USA; 5 School of Veterinary Medicine, Murdoch University, 90 South Street, Perth 6150, Australia; robert.shiel@murdoch.edu.au; 6 Small Animal Clinic, Centre for Clinical Veterinary Medicine, LMU Munich, 80539 Munich, Germany; a.wehner@lmu.de; 7 AniCura Medisch Centrum Voor Dieren, Isolatorweg 45, 1014 AS Amsterdam, The Netherlands; miguel.campos@anicura.nl; 8 Small Animal Department, Ghent University, 9820 Ghent, Belgium; sylvie.daminet@ugent.be; 9 Department of Veterinary Medical Sciences, University of Bologna, 40064 Bologna, Italy; federico.fracassi@unibo.it; 10 Antech Diagnostics, Warwick CV34 6RR, UK; peter.graham@antechdx.com; 11 College of Veterinary Medicine, Michigan State University, East Lansing, MI 48824, USA; korchiaj@msu.edu; 12 Department of Veterinary Clinical Sciences, School of Veterinary Medicine, Louisiana State University, Baton Rouge, LA 70820, USA; plathan@lsu.edu; 13 Hospital Escolar Veterinário, Faculty of Veterinary Medicine, University of Lisbon, 1300-477 Lisbon, Portugal; r.oliveiraleal@gmail.com; 14 Institute of Experimental Biology and Medicine (IBYME)—CONICET, Buenos Aires C1428ADN, Argentina; dmiceli@fvet.uba.ar; 15 Small Animal Clinical Studies, School of Veterinary Medicine, University College Dublin, D04 V1W8 Dublin, Ireland; carmel.mooney@ucd.ie; 16 Veterinary Teaching Hospital, Veterinary School, Complutense University of Madrid, Avenida Puerta de Hierro, S/N, 28040 Madrid, Spain; mdpa@vet.ucm.es; 17 Animal Endocrine Clinic, 21 West 100th St., New York, NY 20025, USA; drpeterson@animalendocrine.com; 18 College of Veterinary Medicine, Cornell University, 602 Tower Rd., Ithaca, NY 14853, USA; 19 Department of Companion Animal Clinical Studies, Faculty of Veterinary Science, University of Pretoria, Onderstepoort Private Bag X04, South Africa; johan.schoeman@up.ac.za

**Keywords:** consensus, terminology, thyroid, hyperthyroidism, hypothyroidism, diabetes mellitus, endocrinology, Delphi

## Abstract

Companion animal endocrinology has benefited from international standardisation of disease terminology for diabetes mellitus, Cushing’s syndrome, and hypoadrenocorticism through Project Agreeing Language in Veterinary Endocrinology (ALIVE). A group of 14 experts and one chair convened for the third cycle of Project ALIVE, focusing on thyroid disease terminology. The cycle employed the modified Delphi approach from previous cycles, augmented by procedural refinements—such as inclusion of an off-site chair and stricter adherence to timelines —to improve efficiency and flexibility. Novel in this round was the integration of feedback from a previous cycle, which resulted in updated definitions for diabetes mellitus originally developed in ALIVE Cycle 1. Outcomes: A 100% consensus was achieved among panellists and 91.4–100% among 105 members of international veterinary endocrinology societies (32% of total memberships) over 78 thyroid-related terminology items and five revised definitions pertaining to diabetes mellitus. These standardised definitions are expected to facilitate clearer communication and education, enhance diagnostic consistency, support research comparability, and improve clinical care in feline and canine endocrine diseases.

## Simple Summary

After having achieved international consensus over disease, diagnosis, classification, and monitoring concepts in the area of companion animal diabetes mellitus, Cushing’s syndrome, and hypoadrenocorticism, a group of 14 experts and one chair embarked on the third cycle of project “Agreeing Language in Veterinary Endocrinology” (ALIVE), this time focusing on thyroid disease terminology. This cycle’s methods followed, like previous ones, a modified Delphi-approach with small changes to improve efficiency and flexibility, including an off-site chair. For the first time, additionally, feedback on definitions of a previous cycle was incorporated, leading to an update of diabetes mellitus related definitions of ALIVE Cycle 1. This third cycle was completed successfully, accomplishing a majority-based consensus among panellists and international veterinary endocrinology society memberships over 78 thyroid related terminology and five updated diabetes mellitus definitions. As has been the case with the definitions created for other hormonal diseases, it is hoped this work will improve education, research, diagnosis, and treatment in cats and dogs with endocrine disease.

## 1. Introduction

The “Agreeing Language in Veterinary Endocrinology” (ALIVE) initiative was launched in 2016 by the European Society of Veterinary Endocrinology (ESVE) and endorsed by the Society of Comparative Endocrinology (SCE). The first two ALIVE cycles successfully established consensus definitions for diabetes mellitus, Cushing’s syndrome, and hypoadrenocorticism in companion animals, producing standardised terminology applicable to research, education, and clinical practice [1,2]. These cycles applied a modified Delphi approach, a structured consensus methodology widely employed in both human and veterinary medicine [1,2,3,4,5,6,7,8,9]. Terminology of these cycles has been frequently employed since [10,11,12,13,14,15,16,17,18,19,20,21,22]. The third cycle of the project took place in 2023 and 2024 and focused on creating consensus over terminology used in the fields of feline and canine hyperthyroidism and hypothyroidism, as well as feedback received on terminology of previous cycles.

## 2. Materials and Methods

### 2.1. Panel Recruitment

**Figure 1:**
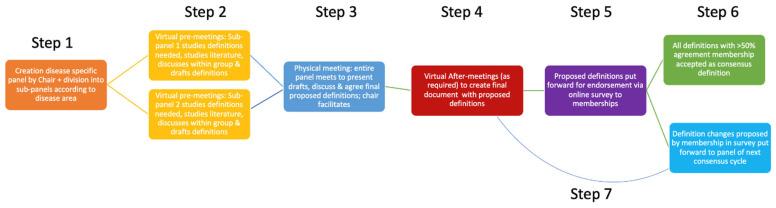
The ALIVE process of Cycle 3 explained in a flow diagram, with step numbers showing the order of steps. ALIVE: Agreeing Language in Veterinary Endocrinology.

A call for participation was distributed to ESVE and SCE members. Fourteen experts were selected by the Chair (SN) to ensure subject-matter expertise, geographical diversity, and a balance of clinical and research perspectives (Step 1, Figure 1). This composition was designed to enhance the representativeness and applicability of the resulting definitions.

### 2.2. Delphi Process

As was the case for cycles 1 and 2, a modified Delphi approach was employed. The process was facilitated by the Chair, who guided discussion and iterative refinement of definitions. On this occasion, the Chair remained off-side for all steps of the process, including the in-person meeting (Step 3, Figure 1). In contrast to previous cycles, additional attention was paid on adhering to a suitable timeline to be able to discuss as many definitions as possible. As in previous cycles, anonymity was not applied, as open dialogue was considered to increase the likelihood of reaching consensus, consistent with previous methodological recommendations [6].

### 2.3. Subdivision and Pre-Meetings

At the outset, the panel was divided into two sub-groups according to professional expertise focusing on either hyperthyroidism or hypothyroidism. Each sub-group appointed a sub-chair (R.S. for hypothyroidism and A.W. for hyperthyroidism) and held a series of virtual pre-meetings to identify, review, and draft preliminary definitions (Step 2, Figure 1).

### 2.4. In-Person Consensus Meeting

Draft definitions were presented by sub-chairs to all panellists and discussed at a two-day in-person meeting, with the Chair remaining off-site (Step 3, Figure 1). During these sessions, definitions were debated, refined, and, where possible, finalised. Definitions requiring further consideration were addressed in follow-up email discussions and virtual meetings (Step 4, Figure 1).

### 2.5. Panel Consensus Criteria

Consensus was defined as agreement by at least 75% of panellists, although unanimous agreement was sought. Definitions not meeting this threshold were revised and re-circulated for further discussion until consensus was achieved.

### 2.6. Membership Endorsement

Panel-approved definitions were submitted for endorsement by ESVE and SCE memberships via an anonymous online survey (Step 5, Figure 1). Members were invited to either endorse or reject each definition and were provided the opportunity to leave comments. Survey invitations were distributed through society mailing lists and promoted via social media and professional meetings. Members were formally reminded of the opportunity to participate in the survey through email on three separate occasions.

A simple majority of respondents (>50%) was deemed sufficient for a definition to qualify as ALIVE-approved terminology (Step 6, Figure 1). Member comments were archived for consideration in future ALIVE cycles (Step 7, Figure 1). A minimum of 20%-member participation was set as a target threshold for this final phase.

### 2.7. Funding and Independence

Project costs were covered by ESVE and external sponsorship. Sponsors were not permitted to influence the design, conduct, or outcomes of the process.

## 3. Results

The 14 panellists achieved 100% agreement over 78 thyroid-associated definitions and five updates to Cycle 1 diabetes mellitus-associated definitions. During Step 5, definitions were assessed by 105 ESVE and SCE members (35% of combined memberships). A simple majority, ranging from 91.4 to 100%, was achieved among the responding memberships. The specific definitions are shown below, including the level of agreement obtained.

### 3.1. Hyperthyroidism

#### 3.1.1. Euthyroidism (Endorsement 101/105)

Normal thyroid function as opposed to hypo- or hyperthyroidism. It can apply to animals without or after successful treatment of thyroid disease.

#### 3.1.2. Hyperthyroidism (Endorsement 100/105)

Excess of thyroid hormone production by the thyroid gland.

#### 3.1.3. Overt Hyperthyroidism (Endorsement 97/105)

Phenomenon characterised by increased circulating concentrations of thyroid hormones (total and free thyroxine [T4]), with evidence of thyroid stimulating hormone (TSH) suppression. Almost all affected cats have clinical signs of thyrotoxicosis and palpable goitre.

#### 3.1.4. Thyrotoxicosis (Endorsement 105/105)

Clinical manifestations that arise from the effects of excess thyroid hormone concentrations secondary to hyperthyroidism or to exogenous sources (e.g., iatrogenic, dietary).

#### 3.1.5. Thyroid Storm (Endorsement 99/105)

There is currently no clear scientific evidence that cats or dogs develop thyroid storm. In humans, thyroid storm is an endocrine emergency characterised by multiple organ failure and hyperthermia caused by severe thyrotoxicosis and is often associated with triggering illnesses and a high mortality rate. Some hyperthyroid cats can be intolerant to stress and can present in cardiac or respiratory distress with or without congestive heart failure and require appropriate management. This does not mean they meet the criteria for ‘thyroid storm’.

#### 3.1.6. Subclinical Hyperthyroidism (Endorsement 97/105)

Some cats with hyperthyroidism are not overtly affected. There may only be subtle clinical signs or a barely palpable goitre with within the reference interval circulating total T4 concentration (usually towards the upper reference interval limit). These cats can be addressed as subclinical and usually have evidence of TSH suppression. Hyperthyroidism can then be confirmed by additional tests (scintigraphy or additional laboratory tests [fT4 via equilibrium dialysis or retest with T4 later]). Caution is advised in starting treatment at this stage.

#### 3.1.7. Primary Hyperthyroidism (Endorsement 103/105)

Hyperfunction of the thyroid gland itself, not caused by stimulation by TSH or thyrotropin-releasing hormone (TRH).

#### 3.1.8. Central Hyperthyroidism (Endorsement 104/105)

Hyperfunction of the thyroid gland caused by excess stimulation by TSH (secondary) or TRH (tertiary). There are currently no reports of these disorders in cats or dogs.

#### 3.1.9. Secondary and Tertiary Hyperthyroidism (Endorsement 104/105)

See central hyperthyroidism.

#### 3.1.10. Iatrogenic Hypothyroidism (Endorsement 104/105)

Hypothyroidism, as defined according to ALIVE criteria, induced by treatment of thyroid disease. Such treatments include thyroidectomy, radioactive iodine, and external beam radiation, which can result in transient or permanent hypothyroidism and anti-thyroid drugs that usually result in transient hypothyroidism.

#### 3.1.11. Exogenous Causes of Thyrotoxicosis (Endorsement 103/105)

Oversupplementation (iatrogenic) or inadvertent ingestion of thyroid hormone medications or consumption of diets containing excess thyroid hormones (e.g., contamination or use of rations containing thyroid tissue [dietary]).

#### 3.1.12. Iatrogenic Thyrotoxicosis (Endorsement 99/105)

Thyrotoxicosis due to oversupplementation of thyroid hormone.

#### 3.1.13. Dietary Thyrotoxicosis (Endorsement 105/105)

Consumption of diets containing excess thyroid hormones (e.g., contamination or use of rations containing thyroid tissue).

#### 3.1.14. Goitre (Endorsement 97/105)

Any enlargement of the thyroid gland. The presence of goitre does not indicate hyper- or hypofunction. In feline hyperthyroidism, terms such as ‘thyroid nodule’ and ‘thyroid slip’ are often used interchangeably to describe palpable goitre.

#### 3.1.15. Thyroid Palpation Technique (Endorsement 96/105)

The classic technique is performed with the cat in a sitting position, the neck extended, and the clinician’s thumb and forefinger sweeping downwards on each side of the trachea from the larynx to the sternal manubrium until the goitre is palpated. The thyroid gland size is scored using a validated semi-quantitative estimation: the scoring system ranges from 0 (non-palpable) to a maximum of 6 (nodule >25 mm), with score 1 = 1–<3 mm, score 2 = 3–<5 mm, score 3 = 5–<8 mm, score 4 = 8–<12 mm, and score 5 = 12–25 mm. If the thyroid glands are not equal in size, thyroid size should be recorded for each nodule.

#### 3.1.16. Ectopic Overactive Thyroid Disease (Endorsement 103/105)

Refers to a condition where hyperfunctioning thyroid tissue is present outside of the thyroid gland. It results from developmental defects at early stages of thyroid gland embryogenesis. It is usually found between the base of the tongue and the base of the heart and occurs in <5% of cases.

#### 3.1.17. Follicular Nodular Goitre (Endorsement 101/105)

Follicular nodular goitre (previously known as multinodular goitre) is the second most common cause of hyperthyroidism in humans. The condition most closely resembling this human condition and commonly observed in older cats is called adenomatous hyperplasia or adenoma.

#### 3.1.18. Graves’ Disease (Endorsement 102/105)

It is a multi-system autoimmune disorder characterised by TSH receptor antibodies and bilateral goitre. It is usually associated with hyperthyroidism. This form of hyperthyroidism is the most common type of hyperthyroidism in humans but has not yet been recognised in cats.

#### 3.1.19. Adenomatous Hyperplasia or Adenoma of the Thyroid Gland (Endorsement 101/105)

Benign, non-cancerous growth of thyroidal epithelial cells. Feline hyperthyroidism is most commonly (approximately 98% of cases) associated with benign adenomatous hyperplasia or adenoma of the thyroid gland (previously referred to as toxic nodular goitre). Left untreated, thyroid tissue in affected cats continues to grow and hyperfunction, resulting in more severe clinical signs over time. This condition in cats most closely resembles follicular nodular disease (previously known as multinodular goitre), which is the second most common cause of hyperthyroidism in humans.

#### 3.1.20. Thyroid Carcinoma (Endorsement 100/105)

Malignant cancerous growth of the thyroidal epithelial cells. The prevalence of thyroid carcinoma causing hyperthyroidism in cats is low, at approximately 2%, where it is the most common cause of hyperthyroidism in dogs. Definitive diagnosis of thyroid carcinoma is best confirmed by documenting distant metastases by diagnostic imaging (preferably scintigraphy). However, in cases in which overt metastatic disease is absent, the diagnosis of carcinoma can be inferred by histopathology. Thyroid carcinomas can be extremely large and may require very high doses of radioactive iodine for successful treatment.

Comments:

- Specific criteria for histopathological characterisation of thyroid carcinoma have been described [23].

#### 3.1.21. SHIM-RAD (Endorsement 103/105)

Hyperthyroid cats that have been managed for many months to years with anti-thyroid drugs can develop resistance to anti-thyroid medication and probable malignant transformation of the thyroid gland. The acronym “SHIM-RAD” is used to characterise this sub-group of cats clinically based on their history, clinical features, and scintigraphic findings. These SHIM-RAD cats are defined on the basis of five characteristics: (1) **S**evere hyperthyroidism (serum T4 > 24 μg/dL or >300 nmol/L); (2) **H**uge thyroid tumour size or volume; (3) **I**ntrathoracic tumour nodule(s); (4) **M**ultifocal disease pattern [≥three nodules]; and (5) **R**esistance to **A**nti-thyroid **D**rug treatment. As in those that present initially with thyroid carcinoma, most of these cats require very high doses of radioiodine (e.g., 30 mCi, 1100 MBq) in order to completely ablate all thyroid tissue.

#### 3.1.22. Risk Factors for Development of Hyperthyroidism (Endorsement 103/105)

A risk factor is a factor in the animal’s environment (extrinsic) or in the animal itself (intrinsic) that may contribute to the development of the disease. In feline hyperthyroidism, multiple factors are thought to contribute to the development of the disease.

#### 3.1.23. Intrinsic and Extrinsic Risk Factors for Development of Hyperthyroidism (Endorsement 103/105)

Signalment, age, sex, and breed are all recognised intrinsic risk factors. Particularly, in feline hyperthyroidism advancing age, sex (female), and breed (non-purebred) are potential intrinsic risk factors. Potential extrinsic risk factors include substances in food, drugs, and the environment. In feline hyperthyroidism, many substances can act as goitrogens (substances that disrupt thyroid hormone production and consequently stimulate TSH release) or as thyroid disruptors (chemicals that interfere with the hypothalamic–pituitary–thyroid axis directly or via thyroid hormone receptors). Disparate dietary iodine content, polybrominated diphenyl ethers (PBDEs), and bis-phenol A are implicated as potential extrinsic risk factors. The level of evidence for other substances (e.g., soy proteins) is low.

#### 3.1.24. Thyroid Scintigraphy (Endorsement 105/105)

Thyroid scintigraphy is a nuclear medicine procedure that produces a visual display of functional thyroid tissue based on the selective uptake of radionuclides by thyroid tissue. Thyroid scintigraphy provides information about both thyroid anatomy and function.

Comments:

- Other imaging modalities, such as ultrasound, computed tomography (CT), or magnetic resonance imaging (MRI), do not provide any functional information but can depict the size and internal structure (gross pathology) of the thyroid gland.

#### 3.1.25. ALIVE Criteria for Diagnosis of Hyperthyroidism—Primary Disease (Endorsement 97/105)

Hyperthyroidism is confirmed by demonstration of increased circulating total T4 concentration in association with supportive signalment and clinical signs.

1. If total T4 is increased in the absence of supportive signalment and clinical signs, reassessment of clinical status (especially if total T4 is clearly increased) or repeat T4 measurement (if total T4 is marginally increased) is recommended.
2. If there are supportive clinical signs, but total T4 is not increased and is in the upper end of the reference interval, hyperthyroidism is possible. In these cases, concurrent non-thyroidal disease can be associated with suppression of total T4 to within reference interval. Subclinical hyperthyroidism is associated with within the reference interval total T4 concentrations. Further diagnostic tests for hyperthyroidism could be considered (demonstration of concurrent suppression of TSH, concurrent increased free T4, increased technetium uptake on thyroid scintigraphy or increased total T4 on repeated testing).

Comments:

- Initial measurement of concurrent total T4 and TSH concentrations may provide more information than total T4 alone.
- Lack of suppression of TSH makes hyperthyroidism unlikely.

#### 3.1.26. ALIVE Criteria for Diagnosis Hyperthyroidism—Central Disease (Endorsement 102/105)

Diagnosis of central hyperthyroidism is achieved by documenting supportive clinical signs and increased circulating total T4 concentration with increased TSH concentration. There may be clinical signs and/or supportive diagnostic imaging findings related to a pituitary tumour.

Comments:

- There are currently no reports of central hyperthyroidism in cats or dogs.

#### 3.1.27. ALIVE Criteria for Diagnosis Hyperthyroidism—Exogenous Disease (Endorsement 102/105)

Diagnosis of hyperthyroidism due to exogenous disease is achieved by documenting supportive clinical signs, increased total T4 concentration, and suppressed TSH concentration. There will also be evidence of an exogenous source and reversal of the abnormalities when the source is withdrawn. Thyroid tissue is not well visualised if thyroid scintigraphy is performed.

#### 3.1.28. ALIVE Criteria for Diagnosis of Iatrogenic Hypothyroidism (Endorsement 102/105)

Diagnosis of iatrogenic hypothyroidism is achieved by documenting hypothyroidism using ALIVE criteria of hypothyroidism, as well as induction by treatment of thyroid disease. Such treatments include thyroidectomy, radioactive iodine, external beam radiation, which can result in transient or permanent hypothyroidism, and anti-thyroid drugs, which usually result in transient hypothyroidism.

Comments:

- Currently, it is challenging to predict whether a cat will be permanently or transiently affected by iatrogenic hypothyroidism after thyroidectomy or radioactive iodine therapy. Nevertheless, a return to euthyroidism is thought likely in most cats and is often seen within one to two years of documenting iatrogenic hypothyroidism. However, a return to euthyroidism has taken more than seven years in some cats.
- Cats with overt hypothyroidism are more likely to have more prolonged or permanent hypothyroidism than cats with subclinical disease. For this reason, clinicians should consider monitoring circulating total T4, TSH, and creatinine concentrations at 1, 3, 6, and 12 months after surgical thyroidectomy or radioactive iodine therapy and then every 6–12 months thereafter. Even if most cats return to euthyroidism in the long-term, thyroid hormone supplementation might help prevent adverse effects of insufficient thyroid hormone concentrations (e.g., worsening azotaemia).
- Specifically, thyroid hormone supplementation should be considered in cats with persistent overt biochemical hypothyroidism (verified by demonstrating low circulating T4 and increased TSH concentrations) on at least two occasions 1–3 months apart, especially with worsening azotaemia (which will develop in over half of cats with overt biochemical hypothyroidism) or in the presence of any supportive clinical signs of hypothyroidism.
- In addition, thyroid hormone supplementation should be considered in cats with persistent subclinical hypothyroidism (verified by demonstrating low reference interval circulating T4 and increased TSH concentrations) on at least two occasions 3–6 months apart. Given the lack of clinical signs in these cats, the decision to start supplementation is primarily based on the development of progressive azotaemia (which can develop in up to a third of these cats).

#### 3.1.29. Iatrogenic Hypoparathyroidism (Endorsement 99/105)

Underactivity of the parathyroid glands, manifesting as hypocalcaemia following thyroidectomy, parathyroidectomy, and other forms of parathyroid gland ablation (heat, ethanol).

ALIVE criteria for a confirmed diagnosis of iatrogenic hypoparathyroidism:

- A history of potential for iatrogenic damage to the parathyroid glands;
- An ionised blood calcium below the reference interval of the methodology used;
- A documented inappropriately low blood PTH.
- Comments:
- The lack of elevation of PTH above the reference interval, despite a below reference interval blood calcium, is considered inappropriate and thus consistent with hypoparathyroidism.
- Given the labile nature of PTH, ALIVE recommends the use of an appropriately validated assay to measure PTH, using recommended sampling, storage, and transportation methods; a laboratory participating in an external quality assurance programme is also recommended.
- A diagnosis of “suspected” iatrogenic hypoparathyroidism could be made without measurement of PTH.
- A diagnosis of “suspected” iatrogenic hypoparathyroidism could be made with documentation of a low total calcium when ionised calcium is not available.

#### 3.1.30. TSH Stimulation Test (Cats) (Endorsement 105/105)

The TSH stimulation test has not been validated for assessment of hypothyroidism in treated hyperthyroid cats. It is also not recommended for the investigation of hyperthyroidism.

#### 3.1.31. TRH Stimulation Test (Cats) (Endorsement 99/105)

Principle: When administered IV to healthy cats, TRH causes consistent increases in serum total T4 concentrations in healthy cats. Cats with hyperthyroidism have autonomous secretion of thyroid hormones (i.e., hormone secretion is independent of pituitary control), which suppresses pituitary TSH secretion, and the post-TRH rise in total T4 is blunted or absent, potentially making the test a useful tool for diagnosis of hyperthyroidism in cats with borderline total T4 concentrations.

Protocol: This test is performed by measuring circulating total T4 concentrations before and 4 h after IV administration of TRH (0.1 mg/kg).

Interpretation: To assess the response of serum total T4 to TRH, relative change (percentage) in total T4 concentration after TRH administration is calculated. Normal cats exhibit >60% increase in total T4. Total T4 increases in cats with hyperthyroidism are <50%. Sick euthyroid cats may have similar results to hyperthyroid cats.

Comments:

- The panel does not use this test but recognises that it can be used in certain situations.
- Administration of TRH often causes severe cholinergic and central nervous system-mediated reactions. Within seconds of being given TRH, cats often exhibit transient but severe salivation, tachypnoea, micturition, nausea, vomiting, and diarrhoea.
- The TRH stimulation test would likely be a good diagnostic test if TSH secretion is evaluated directly. One study shows that TRH will stimulate feline secretion using low doses of TRH in healthy cats [24]. By using these low doses, less side effects are being observed.
- The TRH stimulation as a diagnostic test, giving lower doses and measuring TSH directly should be investigated in hyperthyroid cats, cats with nonthyroidal illness, and clinically normal cats to establish testing protocols and diagnostic guidelines for interpretation.

#### 3.1.32. Triiodothyronine (T3) Suppression Test (Endorsement 103/105)

Principle: Administration of T3 (liothyronine) to healthy cats should suppress pituitary TSH secretion, causing a subsequent decrease in the circulating total T4 concentration. Cats with hyperthyroidism have autonomous secretion of thyroid hormones (i.e., hormone secretion is independent of pituitary control), which suppresses pituitary TSH secretion. Thus, administration of T3 to hyperthyroid cats has no further suppressive effect on circulating total T4 concentration. The T3 suppression test can be useful in distinguishing euthyroid from mildly hyperthyroid cats with borderline resting serum total T4 concentrations.

Protocol: Initially, serum is obtained for the determination of both serum total T3 and total T4 concentrations. Owners are then instructed to administer T3 (liothyronine) the next morning at a dosage of 25 μg given orally, three times daily, for two days. On the morning of day 3, a seventh 25 μg dose should be administered and the cat returned to the hospital so that a second blood sample can be obtained. This blood sample, for measurement of both serum total T3 and total T4 concentrations, should be obtained two to four hours after administration of the seventh dose of liothyronine.

Interpretation: Euthyroid cats demonstrate a marked reduction in the serum total T4 concentration after seven doses of synthetic T3. Cats with hyperthyroidism demonstrate minimal or no decrease. The serum T3 concentration should increase in all cats, in which liothyronine is successfully administered, regardless of the status of thyroid gland function, confirming owner compliance. If the serum total T4 concentration fails to decline in a cat that does not demonstrate an increase in serum total T3 concentration, test results should not be trusted.

#### 3.1.33. TSH Suppression (Endorsement 102/105)

This is defined as a serum TSH concentration that is less than the detection limit (<0.03 mg/mL) when measured by the canine TSH (cTSH) chemiluminescent assay (CLIA), or low (<0.01 ng/mL) when measured by the more sensitive TSH bulk acoustic wave assay (TSH-BAW) [25].

#### 3.1.34. Unmasking CKD (Endorsement 102/105)

A mild increase in serum creatinine is expected (and normal) with effective treatment of hyperthyroidism. In some cases, this means that the serum creatinine evolves from within to above the reference interval; this is referred to as unmasking CKD. In other words, the cat develops post treatment renal azotaemia that was not apparent before treatment of hyperthyroidism.

#### 3.1.35. Treatment Success in Hyperthyroidism (Endorsement 95/105)

The goal is the achievement of euthyroidism. This should be assessed primarily by resolution of reversible clinical and clinicopathological abnormalities associated with the disease whilst avoiding complications as subclinical and clinical hypothyroidism. Total T4 concentrations should ideally be within the lower half of the reference interval because values within the upper half may be associated with persistence of hyperthyroidism. Decreased values should be avoided, as they may indicate hypothyroidism but may be unavoidable in cats with significant concurrent non-thyroidal illness. Concurrent measurement of TSH concentrations is recommended. Undetectable TSH concentrations may support persistence of hyperthyroidism if total T4 concentrations are within the upper half of the reference interval. Increased TSH concentrations support clinical or subclinical hypothyroidism depending on whether the total T4 concentration is below or within the reference interval, respectively.

#### 3.1.36. Medical Treatment Hyperthyroidism (Palliative) (Endorsement 104/105)

Medical treatment is defined as a palliative treatment, which has to be given on a daily basis (oral or transdermal). Usually anti-thyroid drugs (thioureylenes) such as methimazole (thiamazole) or carbimazole that block the synthesis of thyroid hormones through inhibition of thyroid peroxidase are used.

#### 3.1.37. Surgical Treatment Hyperthyroidism (Curative) (Endorsement 99/105)

Surgical treatment refers to thyroidectomy and can be considered a curative treatment. It can be performed unilaterally or bilaterally. Intra- and extracapsular approaches are possible. Bilateral thyroidectomy can result in hypothyroidism and hypoparathyroidism.

#### 3.1.38. Radioactive Treatment Hyperthyroidism (Curative) (Endorsement 105/105)

Use of radioactive iodine (radioiodine) is considered the treatment of choice for hyperthyroidism for many cats. Thyroid hormones and thyroglobulin are the only iodinated organic molecules in the body, so any ingested or injected iodine is taken up by the sodium-iodide symporter of thyroid follicular epithelial cells. Thus, radioactive isotopes of iodine are concentrated in the thyroid gland where their beta-particles exert significant local tissue damage, destroying hyperactive thyroid tissue. Adjacent normal tissue can certainly be destroyed as well, but radioiodine is ideally administered at low doses aimed to achieve euthyroidism. Several methods are used for calculating dose of radioiodine needed to treat cats with hyperthyroidism. Hyperthyroidism resolves in 90–95% of radioiodine-treated cats, but many of these cats will develop either overt or subclinical hypothyroidism.

#### 3.1.39. Diet Management Hyperthyroidism (Palliative) (Endorsement 103/105)

A palliative therapy consisting of the administration of a restricted iodine diet that reduces thyroid hormone production from the thyroid gland.

#### 3.1.40. Heat Ablation, Ethanol Injection Treatment Hyperthyroidism (Endorsement 102/105)

Radiofrequency heat ablation and ethanol injection are potentially curative procedures. The affected thyroid lobe is permanently damaged by injection of ethanol or heat. Due to questionable efficacy and side effects, these treatments are not recommended.

#### 3.1.41. Chemotherapy and External Beam Radiation Treatment for Hyperthyroidism (Endorsement 105/105)

Two treatment options for malignant thyroid tumours. Chemotherapy involves the use of systemic drugs to target and destroy tumour cells, while external beam radiation is a form of local radiation therapy.

#### 3.1.42. TSH Suppression After Removal of Thyroid Carcinoma (Endorsement 100/105)

TSH suppression is a treatment administered to attempt to reduce the risk of recurrence after removal of thyroid tumours of follicular cell origin. Common medications include levothyroxine and liothyronine.

### 3.2. Hypothyroidism

#### 3.2.1. Hypothyroidism (Endorsement 105/105)

Hypothyroidism describes inappropriately reduced function of the thyroid gland that results in a state of thyroid hormone deficiency.

#### 3.2.2. Primary Hypothyroidism (Endorsement 105/105)

Primary hypothyroidism is defined as hypofunction of the thyroid gland that is not caused by a deficiency of TSH or TRH.

Comment:

- Primary hypothyroidism can be due to several processes including immune-mediated destruction, thyroid atrophy, neoplasia, congenital disease, or iatrogenic causes including surgery, drugs, and radiation.

#### 3.2.3. Secondary Hypothyroidism (Endorsement 105/105)

Secondary hypothyroidism is a state of thyroid hypofunction characterised by a lack of TSH production by the pituitary gland and not due to a lack of TRH stimulation.

Comment:

- This can be due to several processes including neoplasia, hypophysitis, or congenital diseases, as well as iatrogenic causes including surgery or radiation.

#### 3.2.4. Tertiary Hypothyroidism (Endorsement 105/105)

Tertiary hypothyroidism is hypofunction of the thyroid gland due to a lack of TRH production by the hypothalamus.

Comment:

- This is poorly described in dogs and not yet reported in cats.

#### 3.2.5. Central Hypothyroidism (Endorsement 104/105)

Central hypothyroidism encompasses secondary or tertiary hypothyroidism or both. This term should be used when secondary and tertiary hypothyroidism are not distinguished.

#### 3.2.6. Congenital Hypothyroidism (Endorsement 105/105)

Congenital hypothyroidism refers to hypofunction of the thyroid gland present from birth. Affected animals may not display clinical signs until later in life. It is caused by abnormal development or function of any part of the hypothalamic–pituitary–thyroid axis.

#### 3.2.7. Genetic Hypothyroidism (Endorsement 102/105)

Genetic hypothyroidism is a form of congenital hypothyroidism that results from a genetic mutation. Although rare, mutations in the T4 peroxidase gene have been described in dogs and cats.

#### 3.2.8. Thyroid Dysgenesis (Endorsement 105/105)

Thyroid dysgenesis refers to a structural developmental defect of the thyroid gland.

#### 3.2.9. Thyroid Dyshormonogenesis (Endorsement 104/105)

Thyroid dyshormonogenesis refers to a developmental or acquired abnormality of thyroid hormone synthesis.

#### 3.2.10. Thyroid Atrophy (Endorsement 102/105)

Primary thyroid disease characterised by replacement of normal parenchymal tissue with adipose or fibrous connective tissue, but without significant inflammatory infiltration. In some cases, this may represent an end stage of lymphocytic thyroiditis. The definitive test for thyroid atrophy is thyroid biopsy. However, this is uncommonly performed. The disease is often assumed by demonstration of primary hypothyroidism without the presence of circulating antibodies against thyroglobulin, T4, or T3. In such cases, other potential causes of primary hypothyroidism must be excluded (including iatrogenic, neoplastic, drug-induced, or congenital causes).

#### 3.2.11. Lymphocytic Thyroiditis (Endorsement 103/105)

Primary thyroid disease characterised by multifocal or diffuse infiltration of the thyroid tissue by lymphocytes, macrophages, and plasma cells. This can lead to progressive destruction of thyroid follicles. The definitive test for lymphocytic thyroiditis is thyroid biopsy. However, this is uncommonly performed. Alternatively, the presence of disease can be inferred by demonstration of circulating antibodies against thyroglobulin or thyroid hormones (T4 or T3). The presence of anti-thyroid antibodies does not provide evidence of thyroid dysfunction.

#### 3.2.12. Neoplasia-Induced Primary Hypothyroidism (Endorsement 105/105)

Hypothyroidism caused by neoplasia destroying normal thyroid parenchyma.

#### 3.2.13. Trauma-Induced Hypothyroidism (Endorsement 104/105)

Hypothyroidism caused by trauma to any component of the hypothalamic–pituitary–thyroid axis.

#### 3.2.14. Silent/Subclinical Thyroiditis (Endorsement 102/105)

The presence of lymphocytic thyroiditis without alteration of thyroid function. This is typically identified as thyroid autoantibody positivity without increased TSH or decreased T4 concentrations or clinical signs. This condition may or may not progress to subclinical/compensating hypothyroidism or clinical hypothyroidism. Lymphocytic thyroiditis may resolve in some dogs.

#### 3.2.15. Subclinical Hypothyroidism (Endorsement 102/105)

Subclinical hypothyroidism is a state of increased TSH concentration without decreased T4 concentration or clinical signs. This state reflects partial destruction of the thyroid gland, often associated with lymphocytic thyroiditis. The absence of clinical signs likely reflects maintenance of adequate T4 secretion from residual thyroid tissue due to increased TSH stimulation. This condition may or may not progress to clinical hypothyroidism.

Comment:

- Increased TSH concentration with reference interval total T4 concentration can also be seen during recovery from illness, following removal of thyroid suppressive medications and in certain illnesses such as primary hypoadrenocorticism.
- T4 autoantibodies may also result in an artefactual increase in T4 concentration to within reference interval (or above).

#### 3.2.16. Myxoedema (Endorsement 105/105)

Myxoedema is the increased deposition of glycosaminoglycans within the dermis and when present is strongly suggestive of hypothyroidism in dogs. This can lead to the clinical appearance of non-pitting oedema and a tragic facial expression. This most commonly affects the head but can involve other areas of the body.

#### 3.2.17. Hypothyroid Crisis (Formerly Known as Myxoedema Coma) (Endorsement 105/105)

Hypothyroid crisis is a rare and potentially fatal complication of hypothyroidism characterised by hypothermia, decreased mentation (potentially progressing to coma), and cardiac or respiratory decompensation. It is often associated with severe concurrent disease.

Comments:

- In human medicine, myxoedema is frequently but not consistently observed. Coma is also uncommon. The underlying pathogenesis is related to decompensated hypothyroidism not myxoedema. As a result, the term hypothyroid crisis is preferred.
- The condition is rare, not well characterised in dogs, and has not been reported in cats.

#### 3.2.18. ALIVE Criteria for Diagnosis of Canine Primary Hypothyroidism (Endorsement 102/105)

A diagnosis of canine hypothyroidism should only be pursued in the presence of supportive clinical or clinicopathological changes. First-line testing should include concurrent measurement of at least total T4 and cTSH concentrations. Hypothyroidism should never be investigated by the measurement of total T4 concentrations alone.

- Decreased total T4 with increased cTSH concentrations are consistent with a diagnosis of primary hypothyroidism (observed in approximately 70% of cases).
- Decreased total T4 with reference interval cTSH concentrations are observed in approximately 30% of dogs with primary hypothyroidism. However, this combination of results can also be observed with non-thyroidal illness, in dogs receiving thyroid suppressive medications, and in some healthy dogs, particularly within certain breeds.

Additional testing (cTSH stimulation testing, thyroid scintigraphy, free T4, thyroglobulin or thyroid hormone autoantibodies, TRH stimulation testing) is recommended in these cases.

Comments:

- The most definitive methods for diagnosis of primary hypothyroidism are the rhTSH stimulation test and thyroid scintigraphy.
- Free T4 (by equilibrium dialysis) is less affected by nonthyroidal illness.
- Thyroglobulin or thyroid hormone autoantibody positivity provides evidence of lymphocytic thyroiditis but not thyroid hypofunction.
- Treatment trials with levothyroxine are not recommended unless clinical suspicion is high despite a lack of supportive diagnostic testing.
- Reference interval or increased total T4 concentrations are seen in a small proportion of dogs with primary hypothyroidism. This is most commonly due to assay interference by T4 autoantibodies. Free T4 measurement by equilibrium dialysis is less affected by these antibodies and may be used to support a diagnosis.
- When possible, thyroid function testing is not recommended in animals with severe non-thyroidal illness or while receiving thyroid suppressive medications. If testing is necessary in such cases, results should be interpreted with extreme caution.

#### 3.2.19. ALIVE Criteria for Diagnosis of Central Hypothyroidism (Endorsement 101/105)

A diagnosis of canine hypothyroidism should only be pursued in the presence of clinical or clinicopathological changes considered consistent with the disease. First-line testing should include concurrent measurement of at least total T4 and cTSH concentrations. Suspicion of central hypothyroidism is increased in dogs with documented pituitary diseases (e.g., neoplasia, hypophysitis, or congenital abnormalities) or following pituitary surgery or radiotherapy. A diagnosis of central hypothyroidism should be considered when first-line testing reveals decreased total T4 and reference interval or undetectable cTSH concentrations. This condition can be difficult to distinguish from primary hypothyroidism with reference interval TSH concentrations and non-thyroidal illness. Free T4 may be useful to distinguish between hypothyroidism and non-thyroidal illness. Additional tests to distinguish between primary and central hypothyroidism may include TRH stimulation testing or neuroimaging if pituitary or hypothalamic pathology is suspected.

#### 3.2.20. ALIVE Criteria for the Diagnosis of Subclinical Hypothyroidism (Endorsement 104/105)

Primary subclinical hypothyroidism is a state of increased TSH concentration with reference interval thyroid hormone concentrations (in the absence of interfering autoantibodies) and absence of clinical signs. This is presumed to represent a stage of thyroid disease, where adequate thyroid reserve capacity prevents the development of overt clinical signs. In some cases, progression to clinical hypothyroidism may occur. TSH concentrations may normalise or remain persistently elevated without apparent progression of disease. Treatment of subclinical hypothyroidism is not recommended at this time. Reference interval total T4 (in the absence of interfering autoantibodies) with increased cTSH concentrations may be consistent with subclinical hypothyroidism, primary hypoadrenocorticism, recovery from nonthyroidal illness, or the response following removal of thyroid suppressive medication.

#### 3.2.21. Non-Thyroidal Illness Syndrome (NTIS) (Endorsement 103/105)

A state of altered serum thyroid hormone concentrations due to concurrent disease not directly caused by the thyroid gland. This syndrome represents a continuum of change that is initially a physiological response to illness.

Comments:

- Use of the term euthyroid sick syndrome is discouraged because thyroid function may be suppressed.
- The syndrome is typically characterised by decreased circulating total T4 concentrations. Total T3 concentrations are also commonly decreased. Free T4 concentrations (by equilibrium dialysis) are typically less affected than total T4 but can be decreased in severe disease or, rarely, increased. TSH concentrations may decrease below the lower limit of quantification of the current assay.
- This is a transient state, provided the underlying cause is removed. The degree of thyroid hormone suppression is proportional to the severity of non-thyroidal illness and may have prognostic significance.
- There is no evidence that thyroid hormone supplementation is beneficial in dogs or cats.
- During recovery from non-thyroidal illness, TSH concentrations may be transiently increased as thyroid hormone concentrations normalise.
- In animals with NTIS, thyroid hormone concentrations are inversely correlated with illness severity. In other words, the lower the TT4, fT4, and T3 concentrations, the more severe the animal’s disease and the higher the risk of death. NTIS is associated with high cytokine and serum cortisol concentrations, both of which have a suppressive effect at various levels within the hypothalamic–pituitary–thyroid (HPT) axis depending on the nature of the illness, acuteness of onset, and the severity of inflammation.

#### 3.2.22. Thyroid Hormone Altering Medication (Endorsement 103/105)

Administration of drugs may alter circulating thyroid hormone concentrations. Mechanisms involved include altered synthesis, secretion, transport, or metabolism of thyroid hormones. This is a transient state if the medication is removed, but suppression of thyroid hormone concentrations can persist beyond discontinuation of the drug. It is generally recommended to wait at least four to six weeks following discontinuation of therapy before performing thyroid function testing. However, this recommendation has limited supporting evidence and likely varies depending on the drug, dose, and duration of therapy.

Comment:

- Most of these medications have a suppressive effect on total T4 concentration.
- These drugs include glucocorticoids, non-steroidal anti-inflammatory drugs, barbiturates, and clomipramine.
- Measurement of free T4 concentration often has limited benefit due to variable effects on T4 binding.

#### 3.2.23. Hypothyroidism-Inducing Medication (Endorsement 105/105)

Hypothyroidism-inducing medications can cause clinical hypothyroidism due to effects on the synthesis of thyroid hormones as an intended or unintended effect. These drugs include methimazole, carbimazole, and potentiated sulphonamides.

#### 3.2.24. Threshold for Waiting After Illness, After Stopping Thyroid-Suppressive Medications Before Testing Thyroid Function (Endorsement 103/105)

The timing after a disease process or insult when thyroid hormones return to normal depends on the degree and chronicity of illness, as well as the dose and duration of drug treatment. More severely and chronically affected dogs, or those receiving higher doses of thyroid suppressive medications for long periods, have more prolonged suppression of thyroid hormone concentrations.

Comments:

- It is generally recommended to wait at least four to six weeks following discontinuation of suppressive drug therapy before performing thyroid function testing. However, this recommendation has limited supporting evidence and likely varies depending on the drug, dose, and duration of therapy.

#### 3.2.25. Successful Treatment of Canine Hypothyroidism (Endorsement 101/105)

The goal of treatment of hypothyroidism is the achievement of euthyroidism. This should be assessed primarily by resolution of reversible clinical and clinicopathological abnormalities associated with the disease.

For once-daily therapy, a total T4 concentration (4–6 h post-pill) within the upper half of the reference interval, or slightly above, is desirable. An optimal target for twice-daily therapy has not been well defined.

Treatment success refers to the resolution of clinical signs with minimal long-term complications and good quality of life. It is achieved when clinical signs are solved and total T4 deficit is reverted.

#### 3.2.26. Transient Hypothyroidism (Endorsement 103/105)

Transient hypothyroidism refers to a temporary abnormality of the pituitary–thyroid axis resulting in high TSH and low T4 concentrations that is not due to primary hypothyroidism. Transient hypothyroidism may be induced by a number of possible causes including a recovery phase from concurrent non-thyroidal illness, untreated hypoadrenocorticism, removal of thyroid suppressive medications, or the administration of drugs that block thyroid hormone synthesis.

### 3.3. Definitions for Thyroid Assessment Tests

#### 3.3.1. Total Thyroxine (Total T4 or TT4) (Endorsement 103/105)

Total T4 refers to both protein-bound and free T4 in the circulation. It is typically measured in serum by immunoassay, although it can also be measured with other methods, including liquid chromatography–mass spectrometry.

Comments:

Measurement of total T4 requires the release of T4 from its binding proteins to facilitate its interaction with the measurement system, usually through the use of an agent such as 8-anilino-1-naphthalene-sulphonic acid (ANS).

With the exception of the uncommonly used liquid chromatography–mass spectrometry (LC-MS/MS) method, all veterinary total T4 analyses are competitive immunoassays reliant on antibodies to capture thyroid hormone and facilitate its detection.

Assays designed to measure total T4 in humans are optimised for higher concentrations than are useful for the diagnosis of hypothyroidism in dogs and cats, requiring adaption or canine/feline optimised assays.

Although the underlying immunoassay principle is the same for all commonly used total T4 assays, there are many signal detection technologies and platforms.· Detection systems include radiation (radioimmunoassay, RIA), chemiluminescence (chemiluminescence immunoassay, CLIA), fluorescence (immunofluorescence assay, IFA), and colorimetric (enzyme-linked immunosorbent assay, ELISA, lateral flow).

Most methods require a step to separate the antibody bound from unbound T4 usually by performing a “wash” step (heterogeneous assays). There are technologies now in use in laboratories (cloned enzyme donor immunoassay [CEDIA], Diagnostic Reagents Inc. [DRI]) and clinics (DRI, surface plasmon enhanced fluorescence [SPEF]) that avoid the wash step (homogeneous assay).

RIA has long been considered the gold standard method, but no detection system is uniformly superior to the others, as each individual assay method, its particular capture antibodies, its design, and optimised measurement range will determine its usefulness for veterinary total T4 measurement. Each method used by laboratories or clinics should be validated or verified as fit for purpose with appropriate and ongoing quality control.

Different assay methods used in veterinary laboratories and clinics yield different results, emphasising the importance of local validation and method specific reference intervals and diagnostic cut-offs.

In dogs, subsets of thyroglobulin autoantibodies (TgAA), such as T3 autoantibodies (T3AA) and T4 autoantibodies (T4AA), can interfere with the immunoassay measurement of their respective hormones. Depending on individual assay design, they can cause falsely higher (results that can potentially increase into or above the reference interval) or lower total T4 results.

It is important to recognise the likely direction of change caused by T4AA in the chosen assay method.

For use in diagnostic decision-making, total T4 results generated in clinics should be subject to method verification, performance evaluation, internal quality control, and external quality assessment in the same way as they would in reference laboratories. Results should be interpreted with caution and repeated in a commercial laboratory particularly if equivocal or unexpected.

#### 3.3.2. Free Thyroxine (Free T4 or fT4) (Endorsement 105/105)

Free T4 refers to the small amount (approximately 0.1%) of T4 in the circulation that is not protein-bound. It is typically measured in serum by immunoassay either with or without a step to separate free from bound T4 in the sample. It can also be measured with other methods, including liquid chromatography–mass spectrometry.

Comments:

- Immunoassays for free T4 do not include 8-anilino-1-naphthalene-sulphonic acid (ANS), and theoretically only unbound T4 is able to interact with detection antibodies.
  There are two distinct methods commonly used for free T4 measurement.
  One method involves equilibrium dialysis to separate the protein-bound from the free T4, followed by an ultrasensitive radioimmunoassay for measurement of T4 in the dialysate. This method is not widely available, subject to stringent sample handling requirements, and is more difficult for routine quality control. Nevertheless, it is the only method currently commercially available that measures true free T4 concentrations capable of providing more diagnostic information than total T4 alone.
  The second method does not include a separation step and is made possible through the use of a labelled T4 analogue that theoretically does not react with thyroid hormone binding proteins and is thus available to compete with free T4 for antibody binding sites.
  Because the protein binding kinetic assumptions underlying analogue assays can be violated in certain physiological and pathological states, analogue free T4 methods may be subject to interference that generates results that do not provide more information than obtained by total T4 measurement. Circumstances that promote the release of T4 from binding proteins such as increased free fatty acid concentrations and furosemide therapy will cause increases in the measured free T4.
  In dogs, subsets of thyroid hormone antibodies (T3AA and T4AA) or anti-thyroid antibodies can interfere with the immunoassay measurement of their respective hormones. Interference is more likely with analogue methods compared with those that utilise equilibrium dialysis for separation of free from bound thyroid hormones. For the analogue methodologies commonly used for free T4 measurement, T4 AAs can cause falsely higher (results that can potentially increase into or above the reference interval) free T4 results. Free T4 results may be affected, with or without a similar effect on total T4 results.

#### 3.3.3. Thyrotropin (TSH) (Endorsement 96/105)

Hormone secreted from the pituitary gland that regulates thyroid activity. Its measurement can aid the diagnosis of thyroid disorders. The pituitary gland constantly monitors circulating concentrations of T4 and T3 and decreases or increases the secretion of TSH, respectively, in response to even the slightest increase or decrease in thyroid hormone concentrations. Therefore, finding a low serum TSH concentration is consistent with hyperthyroidism, even if total or free T4 concentrations remain in the high-normal range (a situation defined as subclinical hyperthyroidism). In contrast, finding a high serum TSH concentration is considered diagnostic for hypothyroidism, even if serum total or free T4 concentrations remain low-normal (a situation defined as subclinical hypothyroidism).

#### 3.3.4. Minimum Canine Hypothyroidism Panel (Endorsement 105/105)

A minimum thyroid diagnostic panel for canine hypothyroidism would include a measure of thyroid gland output (looking for decreased total T4 or free T4 in case of hypothyroidism) and a measure of feedback on thyrotrophs (looking for elevation of TSH in case of primary hypothyroidism).

Comments:

- The additional inclusion of TgAA or T4AA in a profile, if negative, reassures that there is no potential antibody interference in thyroid hormone results.
  The inclusion of free T4 by equilibrium dialysis may assist in correctly identifying the effects of non-thyroidal illness or medications.

#### 3.3.5. Minimum Feline Hyperthyroidism Panel (Endorsement 102/105)

In cats suspected of hyperthyroidism, mostly only TT4 is measured; however, in cats with emerging or mild disease, vague clinical signs, or a thyroid nodule cannot be palpated, the use of free T4 and TSH concentration can be helpful.

Comments:

- Emerging, more sensitive TSH tests might help in the future. A low TSH concentration (<0.01 ng/mL) detected by such sensitive tests supports the diagnosis of hyperthyroidism (example test: pubmed.ncbi.nlm.nih.gov/38382201 [accessed on 4 April 2024]).

#### 3.3.6. T3 and T4 Autoantibodies (T3AA and T4AA) (Endorsement 104/105)

In dogs, T3AA and T4AA, respectively, are circulating markers for inflammatory pathology of the canine thyroid glands. They are subsets of TgAA, such that if a dog is TgAA negative, it will not have T3AA or T4AA. However, the converse is not true; it is possible to be TgAA positive and T3AA and T4AA negative.

Comments:

T3AA can be expected in a higher proportion of hypothyroid dogs than T4AA.

Currently, there are no commercially available assays for their measurement, and those that are available in laboratories will be in-house methods and may provide numerical or titre results that are not directly comparable.

Autoantibodies do not provide an indication of thyroid function, and their sole presence does not provide an indication for treatment.

#### 3.3.7. Thyroglobulin Autoantibodies (TgAA) (Endorsement 104/105)

Thyroglobulin autoantibodies are circulating markers for inflammatory pathology of the canine thyroid gland.

Comments:

- In human thyroiditis, the more commonly detected antibodies are anti-thyroperoxidase, but these are inconsistently found in the canine disease.
  A commercial canine TgAA ELISA is available and widely used.
  In-house-derived methods are also in use.
  Whichever method is used, it is important that steps are taken in the method to mitigate the possibility of misleading results from non-specific binding of non-TgAA antibodies.
  Autoantibodies do not provide an indication of thyroid function, and their sole presence do not provide an indication for treatment.

#### 3.3.8. Total Triiodothyronine (Total T3 or TT3) (Endorsement 103/105)

Total T3 refers to both protein-bound and free T3 in the circulation. It is not the main product of the canine or feline thyroid gland and is mostly generated peripherally. As is the case for total T4, total T3 measurement relies on immunoassay. Its measurement for clinical purposes is not recommended by the ALIVE panel. Total T3 has a much lower diagnostic performance than total T4 for both canine hypothyroidism and feline hyperthyroidism, as it is a less direct measure of thyroid gland function than T4. In addition, a large proportion of hypothyroid dogs (approximately 30%) have TgAA subsets that cross react with T3 (T3AA) that could interfere in total T3 immunoassays and generate misleading results.

An example circumstance in which total T3 has been suggested to be more useful than total T4 is in the attempted diagnosis of hypothyroidism in sighthounds. The total T4 and free T4 reference intervals for sighthounds often extend below reportable range of assays, whereas sighthound reference intervals for total T3 may be more similar to other breeds and be within assay reportable ranges. This is, however, not a universally accepted diagnostic practice, given the lack of evidence in its favour.

#### 3.3.9. Free Triiodothyronine (Free T3 or fT3) (Endorsement 105/105)

Free T3 it is the bioactive form of thyroid hormone, and it is free to interact with tissues. For reasons similar to those for total T3, free T3 is seldomly included in thyroid diagnostic panels. There is a theoretical rationale as to why free T3 would be an ideal assessment of thyroid function. However, in practice, it does not appear to be a good indicator of thyroid gland hormone output.

### 3.4. Updates Definitions Previous ALIVE Cycles

#### 3.4.1. Diabetic Remission (Endorsement 105/105)

A patient previously diagnosed with diabetes mellitus (DM) using ALIVE criteria which shows no evidence of DM according to ALIVE criteria a minimum of four weeks after cessation of anti-diabetes pharmacological therapy (e.g., insulin, sodium glucose co-transporter inhibitor) is considered to have achieved diabetic remission.

Comments:

- The patient would still be considered to be in a state of diabetic remission if the patient continues to receive a specific dietary treatment.

#### 3.4.2. Diabetic Ketoacidosis (DKA) (Endorsement 104/105)

DKA is a potentially fatal metabolic complication of diabetes mellitus. DKA consists of the biochemical triad of hyperglycaemia, ketonaemia or ketonuria, and metabolic acidosis. These patients are, in principle, clinically unwell.

ALIVE criteria for diagnosing DKA:

- Diagnosis of DM according to ALIVE criteria;
- Demonstration of ketonaemia defined as increased beta-hydroxybutyrate concentration, AND/OR ketonuria or ketonaemia, defined as detectable ketones using nitroprusside test strips for ketonuria or ketonaemia;
- Demonstration of metabolic acidosis defined as a venous/arterial blood pH < 7.35 and decreased bicarbonate.

Comments:

- When blood gas analysis is unavailable, a patient that is unwell and meeting the above remaining criteria should be suspected of suffering from DKA.
  Demonstration of ketonuria or elevated blood ketones in an animal that is clinically well means the animal will not be suffering from DKA. Instead, this constitutes “Diabetic Ketosis”.

#### 3.4.3. Diabetic Ketosis (DK) (Endorsement 102/105)

DK consists of the biochemical combination of hyperglycaemia, ketonaemia, or ketonuria, but no metabolic acidosis. These patients are, in principle, clinically well.

ALIVE criteria for diagnosing DK:

- Diagnosis of DM according to ALIVE criteria
- Demonstration of ketonaemia defined as increased beta-hydroxybutyrate concentration, AND/OR ketonuria or ketonaemia, defined as detectable ketones using nitroprusside test strips for ketonuria or ketonaemia;
- Demonstration of absence of metabolic acidosis.

Comments:

- When blood gas analysis is unavailable, a patient which is well and meets the above remaining criteria should be suspected of DK.
  Demonstration of ketonuria or elevated blood ketones in an animal that is clinically well means the animal will not be suffering from DKA.
  There is currently no evidence that diabetic ketosis constitutes a greater risk of future DKA.

#### 3.4.4. Euglycaemic Diabetic Ketoacidosis (eDKA) (Endorsement 103/105)

As per ALIVE definition of DKA, as well as presenting with blood or interstitial glucose concentrations < 14 mmol/L (252 mg/dL).

Comments:

- This condition can be encountered when patients have been receiving sodium glucose co-transporter 2 inhibitor and/or insulin treatment.
  When blood gas analysis is unavailable, a patient which is unwell and meets the eDKA remaining criteria should be suspected of suffering from eDKA.
  Demonstration of ketonuria or elevated blood ketones in an animal that is clinically well means the animal will not be suffering from eDKA. Instead, this constitutes “Euglycaemic Diabetic Ketosis” (eDK).

#### 3.4.5. Euglycaemic Diabetic Ketosis (eDK) (Endorsement 101/105)

As per ALIVE definition of DK, as well as presenting with blood or interstitial glucose concentrations <14 mmol/L (252 mg/dL)

Comments:

- Demonstration of ketonuria or elevated blood ketones in an animal that is clinically well means the animal will not be suffering from eDKA.
  There is currently no evidence that eDK constitutes a greater risk of future (e)DKA.

## 4. Discussion

The third Cycle of Project ALIVE achieved consensus among the panellists and subsequently the majority of responding memberships of the participating veterinary endocrinology societies over 78 thyroid disease-associated terminology concepts. In addition, five diabetes-related terminology concepts were either updated or added in response to feedback on ALIVE Cycle 1. This makes the current cycle the most productive cycle to date.

The increased productivity might in part have been due to the productive nature of the participating individual panel members as individuals and as a team, the topic area, and, likely, also due to the increased experience with the process. Having the same Chair for the process as in Cycle 1 and 2, as well as some overlap of some panel members across the three cycles, might also have contributed to this productivity. On this occasion, the Chair (SN) was guiding the entire process remotely, including Step 3, for the first time. The physical distance might have helped in maintaining oversight over the process, including enabling stricter time management.

Also, for the first time, feedback after the publication of Cycle 1 was provided to the panel of Cycle 3 (Step 7) and assessed by this current panel, and, as a result, updated definitions were put forward to the membership during this current Cycle 3. This led to updated diabetes mellitus ALIVE terminology, which was particularly important given the arrival of a new anti-diabetes pharmacological therapy: sodium glucose co-transport 2 inhibitors. Indeed, patients could have been wrongfully classified as “in diabetic remission” when adhering to the previous remission definition, given that it defined remission as cessation of exogenous insulin therapy. This new definition for remission, as well as (e)DKA and (e)DK, now helps provide clarity in light of situations arising from the introduction of this new drug category. Further adaptations will become necessary as the field of veterinary endocrinology evolves.

As another example of such feedback, during the review process of this publication, it was highlighted that the term “palliative” in the context of medical treatment of hyperthyroidism could be perceived to be inappropriate. The Cambridge Dictionary and UK NICE guidelines would not use this term in this context. Nonetheless, the term was explicitly chosen by the ALIVE panel and endorsed by the veterinary endocrine societies because medical treatment does not take away the cause of hyperthyroidism—the thyroid hyperplasia, adenoma, or carcinoma keeps growing and may eventually become resistant to medical management. Definitions could not be amended as part of the review process of this publication, since the definitions presented are the result of carefully created consensus. Only corrections of grammar, readability, or typos were conducted. Suggestions for corrections can nevertheless be made during the next cycle, after which the next ALIVE panel will consider this change, before the veterinary endocrine societies can choose to adopt such change.

Towards the future, an ongoing regular process of updating previous terminology is envisioned, and Step 7 of the employed modified Delphi-method accommodates this. For this reason, readers are encouraged to regularly visit the continuously updated online bank of ALIVE terminology located on www.esve.org. All those active in the field are encouraged to continue to provide feedback to the Chair (SN) should there be further potential for improvement to the terminology provided by the current, past, and future Cycles of project ALIVE.

The impact of the ALIVE Cycles has already been seen as a result of previous ALIVE cycles. For instance, the often referred to and recently updated international feline diabetes mellitus care consensus guidelines by iCatCare now promote the use of several ALIVE definitions generated during ALIVE Cycle 1 for the process of diagnosis, treatment, and monitoring of this disease [22]. Specifically, treatment of diabetes mellitus is now promoted to be primarily guided by the ALIVE Diabetic Clinical Score. At the conclusion of this current Cycle 3, clinicians, researchers, and educators are once again encouraged to continue to use the ALIVE definitions of all ALIVE cycles, helping ALIVE’s mission to improve standards and comparability of research, education and clinical efforts in veterinary endocrinology, and veterinary medicine at large.

## Data Availability

The original contributions presented in this study are included in the article. Further inquiries can be directed to the corresponding author.

## Abbreviations

The following abbreviations are used in this manuscript:

ALIVE	Agreeing Language in Veterinary Endocrinology
ESVE	European Society of Veterinary Endocrinology
SCE	Society of Comparative Endocrinology
